# The association between homocysteine and bacterial vaginosis: results from NHANES 2001–2004

**DOI:** 10.1038/s41598-023-45494-5

**Published:** 2023-12-04

**Authors:** Jing Luo, Tong Chen, Yue Chen, Ze-Min Huang, Xiu-Juan Li, Hao-Kai Chen, Yi-Qi Huang, Xu-Guang Guo

**Affiliations:** 1 Department of Clinical Laboratory Medicine, Guangdong Provincial Key Laboratory of Major Obstetric Diseases; Guangdong Provincial Clinical Research Center for Obstetrics and Gynecology, The Third Affiliated Hospital of Guangzhou Medical University, Guangzhou, 510150 China; 2 Department of Clinical Medicine, The Third Clinical School of Guangzhou Medical University, Guangzhou, 510180 China; 3 Department of Anesthesiology, The Second Clinical School of Guangzhou Medical University Guangzhou, Guangzhou, 510260 China; 4 Guangdong Provincial Key Laboratory of Major Obstetric Diseases, The Third Affiliated Hospital of Guangzhou Medical University, Guangzhou, 510150 China; 5 Key Laboratory of Reproduction and Genetics of Guangdong Higher Education Institutes, The Third Affiliated Hospital of Guangzhou Medical University, Guangzhou, 510150 China; 6 Guangzhou Key Laboratory for Clinical Rapid Diagnosis and Early Warning of Infectious Diseases, KingMed School of Laboratory Medicine, Guangzhou Medical University, Guangzhou, 511436 China

**Keywords:** Biomarkers, Diseases, Health care, Medical research, Pathogenesis, Risk factors

## Abstract

Although no study has directly shown the relationship between bacterial vaginosis (BV) and homocysteine (HCY), we still found some association between these two through extensive literature and data analysis. BV score was calculated by Nugent’s method, less than equal to 6 is negative and greater than equal to 7 is positive. This article describes interrelationships we mined from data extracted by NHANES regarding BV and HCY under multiple covariates. We used two cycles of NHANES 2001–2002 and 2003–2004 in our study. We included 2398 participants in our study who recently completed the interview and the MEC tests. By investigating the relationship between BV and HCY under multivariate conditions, multiple linear regression analysis was performed. These factors may have influenced the results, such as ethnicity, age, education level, body mass index (BMI), etc. Serum vitamin B12, ferritin, percentage of segmented centrioles, and number of segmented centrioles were selected as potential covariates in our study. We observed that both the coarse model and the two adjusted models showed a high correlation between HCY and BV, and the correlation was positive. In the coarse model, OR = 1.26, 95% confidence interval (CI) 1.10, 1.44, *P* = 0.0018); HCY was positively correlated with BV (OR = 1.19, 95% confidence interval (CI) 1.05, 1.34, *P* = 0.0121). Multiple linear regression analysis was used to investigate the connection between BV and HCY under multivariate settings. The results of this study indicate that HCY is positively associated with the prevalence of BV and may play an important role in the prevention and management of BV.

## Introduction

BV is a disease caused by an imbalance in the vaginal microbiota and is more common in women of childbearing age. BV is characterized by a decreased abundance of lactobacilli and an increased abundance of microbial flora, such as anaerobes. By 2004, the prevalence of BV in the US population had reached 29.2%^1^. BV confers increased susceptibility to sexually transmitted infectious diseases, including human immunodeficiency virus (HIV)^2^, as well as several adverse pregnancy outcomes, including preterm birth^3^. There are currently many risk factors for BV, such as a history of sexual life, vaginal flushing, and age^4^. At present, the diagnosis of BV is often made with a Nugent score. The vast majority of scientific research evidence suggests that treatment is recommended for symptomatic women. Although a few studies have shown support for the treatment of asymptomatic BV, most reviews do not recommend screening and treatment of this condition and therefore advocate the treatment of symptomatic women. Numerous epidemiological and microbiological data have shown that BV is a sexually transmitted infection, and it is unclear whether asymptomatic BV is milder than symptomatic BV and whether their pathogenesis, response to antibiotic treatment or complication rates differ. Because of the paucity of data, controversy exists as to whether asymptomatic women with BV should be treated^5^. Thus despite the high prevalence of BV, national guidelines currently do not recommend screening or treatment of asymptomatic bv in women, possibly due to the lack of rigorous clinical trial data demonstrating significant benefit (i.e., adverse outcomes in reducing teenage infections) The common symptoms of BV are vaginal itching, vaginal odor, and so on. The treatment of BV is usually effective. The initial cure rate in one month is 80–90%, but the recurrence rate is also as high as 58%. Therefore, we should pay attention to the disease development process of BV, effectively control the progress of BV, and protect women's health. It is currently believed that lactobacilli in the vagina are able to produce hydrogen peroxide to prevent colonization by associated pathogenic flora, while the role of *inert* lactobacilli in the vaginal environment is controversial^6^.

A thiol-containing amino acid named HCY is primarily metabolized from methionine. The HCY concentration is primarily controlled by two methods: methylation to form methionine or trans sulfurization to form cysteine, and hydrogen sulfide is produced at the same time. HCY can accumulate under different conditions, including genetic factors, diet, lifestyle, and drugs. The deficiency of folic acid, vitamin B12 and vitamin B6, as well as the decrease in related enzyme activity, can inhibit its decomposition, thus increasing the concentration of HCY in cells^7^. The significant absence of these factors is common in elderly individuals, so HCY will increase with age^8^. HCY is present in plasma at normal concentrations between 5 and 15 µmol/L.^9^ Elevated HCY levels are known as hyperhomocysteinemia (HHCY) and confer an increased risk of neurological, cardiovascular, and other diseases^10^. HHCY is thought to be a separate risk factor for cardiovascular disease and atherosclerosis.

Although vaginal lactobacilli rely somewhat on cysteine^11^, HHCY may be a risk factor for BV. Meanwhile, several studies have shown that estrogen can reduce the level of HCY, which may be indirectly through the influence on the expression of related genes or directly affect the synthesis of HCY by influencing the utilization of folic acid in women, thus causing HHCY and becoming a risk factor for BV^12^. HCY may increase superoxide anion release and hydrogen peroxide production by neutrophils, and HHCY may cause excessive superoxide in the body, resulting in lactoferrin in the vagina with insufficient antioxidant capacity to resist, causing an altered intravaginal environment^13^. In this study, we aimed to explore the existing relationship between HCY and BV to further advance the research progress of BV.

## Materials and methods

### Data source

We used two phases, 2001–2002 and 2003–2004, from The National Health and Nutrition Examination Survey (NHANES) for this study. The health and nutrition status of diverse American populations is assessed through the NHANES, a cross-sectional study. Among them, demographic, socioeconomic, educational, age, dietary, and health-related issues were obtained using questionnaire forms, whereas most of the physical examinations, including laboratory results such as HCY levels in this study, were obtained at the Mobile Inspection Center (MEC). Written informed consent was obtained from all participating individuals in this study, which was approved by the research ethics review board of the NCHS (https://wwwn.cdc.gov/nchs/nhanes/default.aspx). We included a total of 21,161 data points, 16,360 after deleting 4801 data points without specific Hcy values, 2557 after deleting 13,803 data points with unknown BVs, and 2398 after excluding 159 data points with unknown covariates.

### Measurement of homocysteine

The Abbott HCY assay was used on the Abbott AxSYM analyzer to measure total HCY (THCY) in plasma using the Fluorescence Polarization Immunoassay (FPIA) from Abbott Diagnostics. Dithiothreitol (DTT) with albumin and other small molecules for free thiols, in contrast to the FPIA detection system, which is made up of certain monoclonal antibodies and fluoresceinated SAH analog tracers, the injection of S-adenosylhomocysteine (SAH) hydrolase catalyzes the conversion of HCY to SAH in the presence of adenosine. THCY was calculated by the Abbott AxSYM immune analyzer using a machine-stored calibration curve. Since the FPIA method is equivalent to others, it was used as the primary method for THCY determination in nhanes 2003–2004. HHCY was defined as plasma total HCY > 15 μmol/L.

### Measurement of bacterial vaginosis

Vaginal swabs were self-collected in a private bathroom at the Mobile Inspection Center (MEC) after participant consent. NHANES personnel collected samples onto slides for Gram staining, after which slides were scanned at low magnification to locate epithelial cell populations. The average number of *Lactobacillus* morphotypes, *Gardnerella* spp., *anaerobic gram-negative rods*, and *Mobil morphotypes* was quantified. The BV fraction was measured by Nugent's method. There are three categories of BV outcome, with Nugent's scores of 0–3 suggestive of normal vaginal flora, 4–6 suggestive of intermediate, and 7–10 suggestive of BV positivity.

### Covariates

These factors may have influenced the results, such as demographic variables including age, race, education level, body mass index (BMI), and other laboratory indicators. Serum vitamin B12, ferritin, percentage of segmented centrioles, and number of segmented centrioles were selected as potential covariates in our study. Participant age was obtained by questionnaire according to certain standards. Race/ethnicity was categorized as non-Hispanic white, non-Hispanic black, Mexican American, other Hispanic, and other races. Educational attainment was categorized into three categories: less than high school, high school, college and above. BMI was determined according to the NHANES III anthropometric procedures standards with the correct technique for participant height and weight, obtained as weight in kilograms divided by height in meters (low BMI, underweight and healthy weight ≤ 24.9, high BMI, overweight and obese > 25). The Quantaphase II Folate/Vitamin B12 radiometric test kit from Bio-Rad laboratory was used to obtain serum vitamin B12. To inactivate endogenous folate binding proteins, serum or whole blood hemolytic samples were combined with 125 folic acid and 57 vitamin B12 in a solution containing dithiols (DTT) and cyanide. The combination was then heated, converting various forms of vitamin B12 to cyanocobalamin. Binding to the immobilized affinity purified porcine intrinsic factor and folate binding protein after waiting for the mixture to cool. The addition of these substances allows the pH of the reaction mixture to be adjusted and buffered to 9.2. The reaction mixture was then incubated at room temperature for one hour. The reaction mixture was centrifuged, decanted and labeled, and unlabeled vitamin B12 was precipitated at the bottom of the tube after binding to immobilized binding proteins. Supernatants containing unbound vitamin B12 were discarded, and the radioactivity associated with the pellets was measured. A standard curve (created from a precalibrated folate/B12 standard in a human serum albumin base) was used to determine the amount of vitamin B12 in each participant's serum. Using a Roche/Hitachi 912 clinical analyzer, ferritin was quantified by immunoturbidimetry. Antigen/antibody complexes are produced when latex-bound ferritin antibodies interact with antigens in the sample. Measurements were made turbidimetrically after agglutination. Measured at 700 nm (primary wavelength), the complexes formed were directly proportional to ferritin concentration. Segmented neutrophil percentages and segmented neutrophil numbers were obtained using a Beckman Coulter maximum instrument in flow check centers (MECs), performing complete blood counts on blood specimens and providing blood cell distribution and differential analysis of white blood cells using VCS technology. These covariates are all available in NHANES with relevant access methods and laboratory test data.

### Statistical analysis

All correlation analyses were performed using the statistical software R (http://www.R-project.org , R Foundation) and using freeware version 4.0 (licensed). We used the weights recommended by the NHANES database, taking into account the oversampling of minority groups, and all included data were analyzed after weighting to achieve as unbiased and accurate effect estimates as possible. In the study population description baseline tables, continuous variables are presented using survey weighted means (95% CI), and *P* values are measured using survey weighted linear regression; Categorical variables used survey weighted percentages (95% CI), and *P* values were measured using survey weighted chi square tests. The relationship between HCY and BV was assessed by input type linear regression models. The standardized β values were used to compare the relative predictive strength of different covariates in the regression model, and the variance inflation factor (VIF) was used to assess multicollinearity for all covariates in the regression model. Covariates were included in the final adjusted model as potential confounders if they changed the estimate of HCY with BV by more than 10% or had a clear association with BV. The log likelihood ratio test was similarly used in threshold effect analysis to assess the linearity of the HCY and BV models. The linear relationship of HCY and BV was further explored using smooth curve fitting. To investigate the quantitative relationship between HCY levels with BV, we performed a linear regression relationship and constructed two adjusted models according to covariates. Hierarchical regression analysis was used to account for differences between age, race, education, and BMI. A two-tailed *P* < 0.05 was considered statistically significant.

### Ethics statement

The authors are accountable for all aspects of the work in ensuring that questions related to the accuracy or integrity of any part of the work are appropriately investigated and resolved. The study was conducted in accordance with the Declaration of Helsinki (as revised in 2013). All information from the NHANES program is available and free for public, so the agreement of the medical ethics committee board was not necessary.

## Results

### Baseline characteristics of the study participants

**Figure 1:**
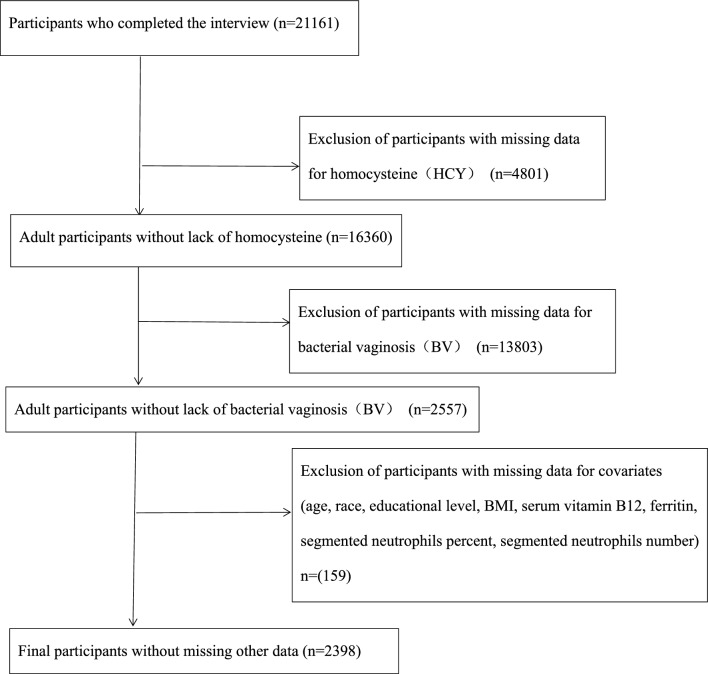
Flow chart for inclusion and exclusion of participants.

We used two cycles of NHANES 2001–2002 and 2003–2004 in this study. We identified 21,161 participants in our study who recently completed the interview and the MEC assessment. Participants with missing data on HCY (n = 4801) and BV (n = 13,803) were excluded. After excluding participants with missing data for age, race, educational level, BMI, serum vitamin B12, ferritin, segmented neutrophil percentage, and segmented neutrophil number, our analysis included 2398 participants. The flowchart of the exclusion criteria is shown in Fig. 1.

The baseline characteristics of the participants are shown in Table 1. Based on Nugent's scores, populations were divided into two categories, where Nugent-BV < 7 was classified as negative and Nugent-BV > 7 as positive. We found that the BV-positive population was more likely to have high BMI, high HCY, low RBC folate, low serum folate, high ferritin, and low segmented neutrophil percentage. No significant differences were found in age, serum vitamin B12 or segmented neutrophil number (*P* > 0.05).

**Table 1:** Baseline characteristics of participants (N = 2398).

Characteristic	Bacterial vaginosis (BV)		*P* value
	Negative (Nugent-BV ≤ 6)	Positive (Nugent-BV ≥ 7)	
N	1353	1045	
Age (year)	31.93 (30.88 ,32.97)	32.82 (31.87 ,33.76)	0.1632
BMI (kg/m^2)	26.66 (26.25 ,27.07)	28.64 (27.88 ,29.41)	< 0.0001
Homocysteine (umol/L)	6.81 (6.64 ,6.98)	7.34 (7.12 ,7.57)	0.0001
Folate, rbc (ng/ml rbc)	283.11 (273.53 ,292.70)	257.09 (248.69 ,265.50)	< 0.0001
Vitamin b12, serum (pg/ml)	513.38 (466.67 ,560.08)	506.07 (472.56 ,539.58)	0.7831
Folate, serum (ng/ml)	13.94 (12.95 ,14.92)	11.90 (11.36 ,12.44)	0.0004
Ferritin (ng/ml)	52.84 (47.88 ,57.80)	64.05 (59.63 ,68.46)	0.0034
Segmented neutrophils percent (%)	60.31 (59.84 ,60.78)	59.23 (58.40 ,60.06)	0.0253
Segmented neutrophils number	4.57 (4.48 ,4.66)	4.66 (4.50 ,4.82)	0.4345
Race			< 0.001
Mexican American	8.52 (6.50 ,11.09)	10.62 (7.37 ,15.06)	
Other hispanic	4.69 (2.68 ,8.06)	7.14 (4.61 ,10.88)	
Non-hispanic white	73.68 (68.91 ,77.95)	53.22 (46.44 ,59.89)	
Non-hispanic black	8.65 (6.61 ,11.24)	23.40 (18.72 ,28.83)	
Other race—including multi-racial	4.47 (3.29 ,6.04)	5.63 (3.37 ,9.25)	
Education level			0.0011
< High school	23.57 (20.46 ,26.99)	27.78 (24.22 ,31.65)	
High school	19.87 (17.70 ,22.23)	24.87 (21.56 ,28.51)	
> High school	56.56 (53.22 ,59.85)	47.35 (43.26 ,51.47)	
Hyperhomocysteinemia(HHCY)			0.0006
No	99.39 (98.68 ,99.72)	97.30 (95.39 ,98.43)	
Yes	0.61 (0.28 ,1.32)	2.70 (1.57 ,4.61)	

*BMI was calculated as the body weight in kilograms divided by the square of the height in meters.

Data in the Table 1, for continuous variables: survey-weighted mean (95% CI), *P* value was by survey-weighted linear regression (svyglm) ; for categorical variables: survey-weighted percentage (95% CI), *P* value was by survey-weighted Chi-square test (svytable).

Based on HCY concentrations, higher than 15 μmol/L were defined as HHCY. We divided participants into two categories, the HHCY population and the non-HHCY population, and the baseline characteristics of participants are shown in Table 2. We found that the HHCY population was more likely to be elderly women with low RBC folate, low serum vitamin B12, low serum folate, high ferritin, and a higher proportion of BV-positive people. No significant differences were found in BMI, serum vitamin B12 or segmented neutrophil count (*P* > 0.05).

**Table 2:** Baseline characteristics of participants (N = 2398).

Characteristic	Hyperhomocysteinemia (HHCY)		*P* value
	No	Yes	
N	2370	28	
Age (year)	32.16 (31.35, 32.96)	41.14 (38.67, 43.60)	< 0.0001
BMI (kg/m^2)	27.47 (27.01, 27.93)	27.68 (24.02, 31.34)	0.9102
Homocysteine (μmol/L)	6.82 (6.70, 6.94)	20.68 (16.24, 25.11)	< 0.0001
folate, rbc (ng/ml rbc)	273.78 (265.21, 282.35)	223.30 (189.85, 256.75)	0.0109
vitamin b12, serum (pg/ml)	511.56 (476.33, 546.79)	357.71 (287.67, 427.76)	0.0007
folate, serum (ng/ml)	13.27 (12.57, 13.97)	6.00 (3.92, 8.09)	< 0.0001
ferritin (ng/ml)	55.61 (52.33, 58.89)	169.75 (74.49, 265.01)	0.0266
segmented neutrophils percent (%)	59.90 (59.43, 60.37)	64.05 (56.84, 71.26)	0.2665
segmented neutrophils number	4.61 (4.54, 4.68)	4.97 (3.91, 6.03)	0.5186
Race			0.9581
Mexican American	9.39 (6.98, 12.51)	7.05 (2.07, 21.41)	
Other hispanic	5.70 (3.82, 8.42)	3.69 (0.31, 32.17)	
Non-hispanic white	65.90 (60.70, 70.74)	66.95 (40.06, 86.00)	
Non-hispanic black	14.09 (11.19, 17.59)	15.59 (5.40, 37.40)	
Other race—including multi-racial	4.93 (3.64, 6.65)	6.72 (0.83, 38.33)	
Education level			0.3660
< High school	25.14 (22.42, 28.08)	34.77 (12.33, 66.89)	
High school	21.84 (20.16, 23.63)	29.88 (9.52, 63.32)	
> High school	53.01 (50.07, 55.93)	35.34 (15.62, 61.76)	
Bacterial Vaginosis			0.0006
Negative	61.18 (58.51, 63.78)	25.83 (9.75, 52.90)	
Positive	38.82 (36.22, 41.49)	74.17 (47.10, 90.25)	

*BMI was calculated as the body weight in kilograms divided by the square of the height in meters;

HHCY: HCY concentration in plasma > 15 μmol/L.

Data in the Table 2, for continuous variables: survey-weighted mean (95% CI), *P* value was by survey-weighted linear regression (svyglm) ; for categorical variables: survey-weighted percentage (95% CI), *P* value was by survey-weighted Chi-square test (svytable).

### The analyses of a linear relationship

We selected these confounders based on their association with the outcome of interest or a change in effect estimate of more than 10%. Supplementary Table S1 shows the association of each confounder with the outcome of interest.

**Figure 2:**
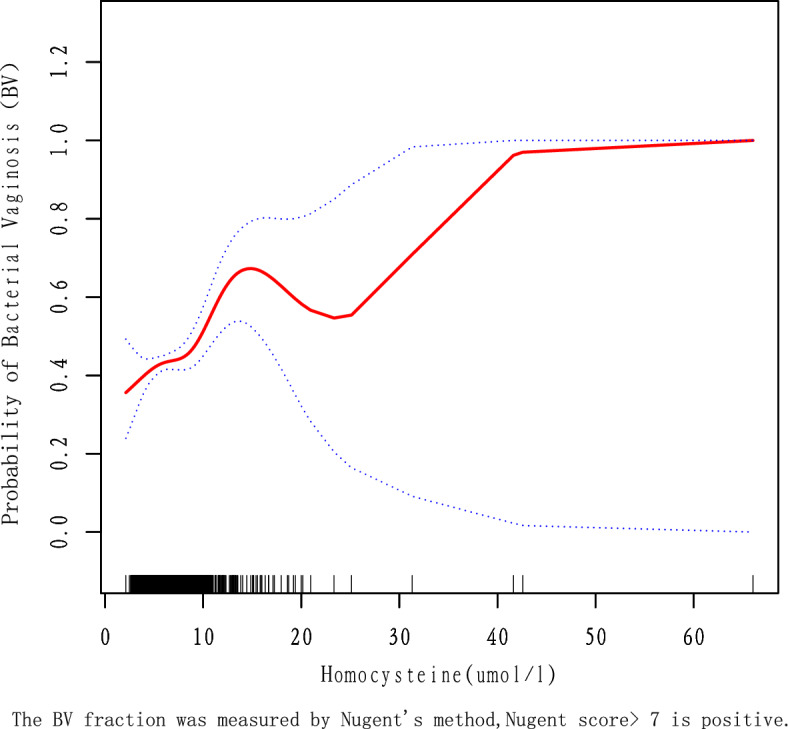
Association of Homocysteine with probability of bacterial Vaginosis, adjusted by model 2.

**Figure 3:**
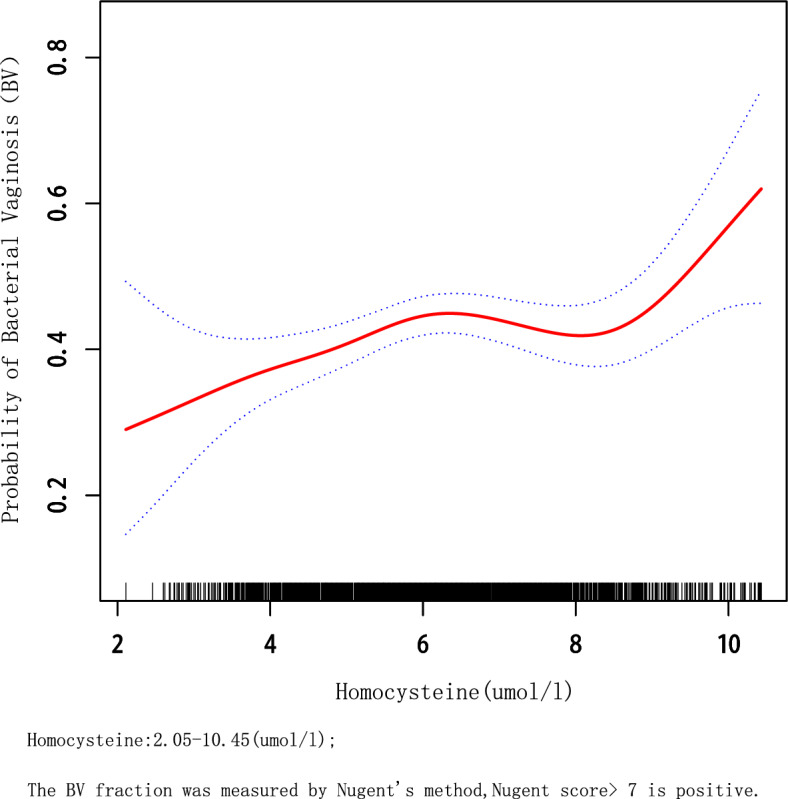
Assocication of Homocysteine with Probability of bacterial Vaginosis after deletion of outliers, adjusted by model 2.

Since HCY is a continuous variable, it is necessary to analyze its linear relationship. In this study, we used a threshold effects model analysis, and based on a log-likelihood ratio test (*P* > 0.5), we concluded that Model 1 better reflected the relationship between HCY and BV, which is described as linear (Supplementary Table S2). Furthermore, the smoothed curve fit was able to capture the overall trend of the relationship (Fig. 2). However, there were some outliers for HCY in the included data samples and it is generally accepted that < Q1 − 1.5*IQR or > Q3 + 1.5 * IQR can be treated as outliers. Q1–Q3 for HCY was 5.2–7.3 (µmol/L) and the final HCY included ranged from 2.05 to 10.45 (µmol/L), it was found that the relationship between HCY and BV remained linear (adjusted for age, education level, race, BMI, serum folate, and erythrocyte folate) and that HCY was positively associated with BV (Fig. 3).

### Linear regression relationship for HCY and BV in models

As shown in Table 3, when regression analysis was performed with HCY levels as a continuous variable, a positive association between HCY and BV prevalence was observed in all models. In the original model, HCY levels were positively associated with BV prevalence (OR = 1.26). A considerable hazard ratio (OR = 1.19) remained in the fully adjusted model. Thus, for every one standard deviation increase in HCY, participants had a 19% increased risk of developing BV (OR = 1.19, 95% CI 1.05, 1.34), which remained statistically significant (*P* = 0.0121).

**Table 3:** Linear regression relationship for Homocysteine(HCY) and Bacterial Vaginosis(BV) in models.

Outcome	Crude model		Model 1		Model 2	
	OR(95%Cl)	*P* value	OR(95%Cl)	*P* value	OR(95%Cl)	*P* value
HCY per SD	1.26 (1.10, 1.44)	0.0018	1.26 (1.10, 1.44)	0.0030	1.19 (1.05, 1.34)	0.0121

For Bacterial Vaginosis (BV): survey-weighted OR (95% CI) *P* value.

Homocysteine (µmol/L) Mean (SD) Median (Q1–Q3): 6.5 (2.7) 6.2 (5.2–7.3).

Nonadjusted model adjusted for None.

Model 1 adjusted for age, educational level, race, and BMI.

Model 2 adjusted for Model 1 + rbc folate, serum folate.

### Stratified analysis

**Figure 4:**
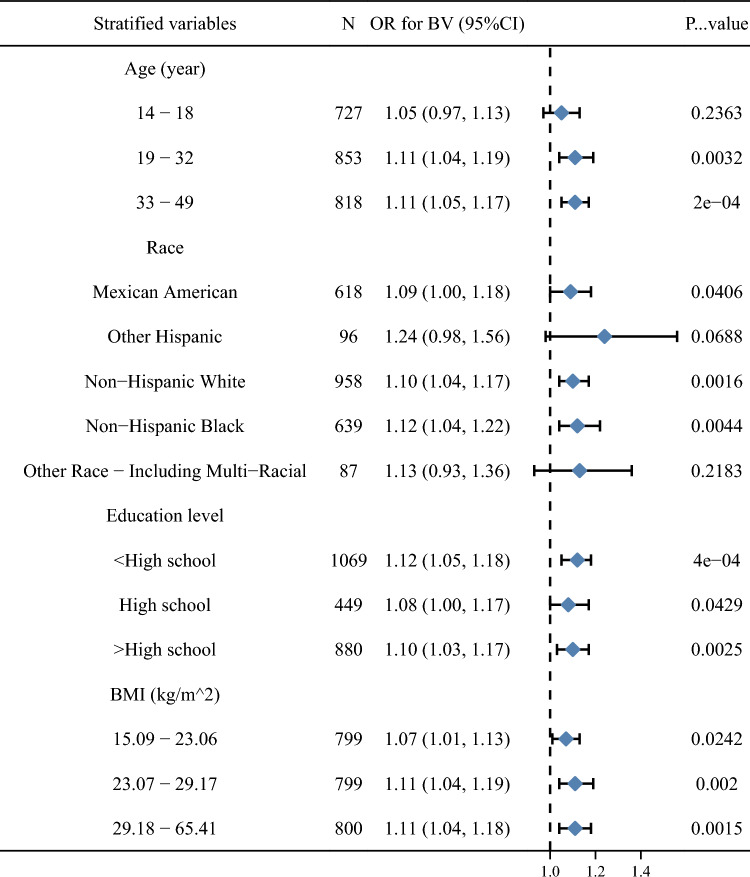
Linear regression relationship for Homocysteine(HCY) and Bacterial Vaginosis(BV) in stratification analysis.

We analyzed stratification according to age, race and education level. The results of the stratified analysis are shown in Fig. 4, where the positive correlation between HCY and BV showed broad agreement in the Mexican American, non-Hispanic white, and non-Hispanic black populations.

## Discussion

This study analyses NHANES data from 2001 to 2004 and elucidates for the first time the association between HCY and BV in adult women in the United States based on epidemiological studies. Our findings suggest that HCY is positively associated with the risk of BV after adjusting for confounding factors. Subsequently, we conducted stratified analyses based on demographic variables and BMI to explore the stability of the association across populations. Although the correlations were not significant in other Hispanic and other races (*P* > 0.5), this may be due to the inclusion of too small a sample in both populations.

The nonprotein amino acid HCY, which contains sulfhydryl, is a metabolic intermediate created in vivo by the demethylation of methionine (Met). HCY is physiologically important for activities including cell cycle progression and the preservation of cellular homeostasis^14^. Plasma concentrations of HCY range from 5 to 15 μmol/L in healthy individuals to as high as 500 μmol/L in patients with HHCY. High HCY levels can cause osteoporosis and ocular lens dislocation, and cardiovascular complications are strongly associated with high HCY levels. Recent studies have demonstrated that elevated plasma HCY is also the root cause of diabetes, Down's syndrome, megaloblastic anemia, and neurological diseases such as Alzheimer's, Parkinson's, and dementia. HCY levels have also been shown to be strongly associated with cancer in recent years^15^. To date, no scholar has studied the relationship between plasma HCY and BV, and there are also no relevant studies explaining the relationship between HCY and BV. We have searched a large body of literature to provide an explanation for this.

BV is a common vaginal infection caused by the replacement of normal *Lactobacillus* spp. by large numbers of anaerobic bacteria^16^, which causes discharge, odor and irritation^17^. Lactobacilli are the main bacterial species capable of preventing parthenogenic and specialized anaerobic bacteria from exceeding their numbers in the vaginal microbiota, thus maintaining healthy microbial homeostasis in the vagina^18^. There is competition between lactobacilli for nutrients in the vaginal epithelium, as well as competition for survival with other bacteria^18^. Additionally, glycogen deposits in the human vagina are under the influence of estrogen. α-Amylase, the enzyme that degrades glycogen into maltose, maltotriose and α-dextrin, is also present^7^, and *Lactobacillus* uses these glycogen breakdown products to produce lactic acid, which acidifies the vagina to a pH of 3.0–4.5, thereby inhibiting the growth of other bacteria^19,20^. In addition, *Lactobacillus* can produce antimicrobial substances to inhibit the growth of several microorganisms^21,22^. Maintaining a high number of resident lactobacilli is an effective marker of female health and good protection against pathogens that cause sexually transmitted infections (STIs)^18^.

More than 20 species of *Lactobacillus* vaginalis have been reported, while four *Lactobacillus* species, *Lactobacillus* curvatus (L. crispatus), *Lactobacillus* griseus (L. gasseri), *Lactobacillus* inserts (L. iners), and *Lactobacillus* jensenii, are the most common in the female vaginal flora^23^. HCY is a major player in methionine synthase (MS), and the coenzyme vitamin B12 is involved in the synthesis of methionine and tetrahydrofolate with 5-methyltetrahydrofolate. l-Methionine is degraded through the transsulfuration pathway to form l-cysteine, which has been shown to support the growth of multiple inert *Lactobacillus* strains, which is not consistent with our findings.

In addition, epithelial cells and immune cells promote homeostasis in vivo by producing anti-inflammatory cytokines such as IL-1RA^24^. In a rat model of PD, HCY creates oxidative stress in the nigrostriatal pathway and decreases the activity of mitochondrial complex I. This causes an increase in the production of hydroxyl radicals, a decrease in glutathione levels, and an increase in the activity of antioxidant enzymes such as superoxide dismutase and catalase^25^. HCY induces excitotoxic effects in cells expressing NMDA-like glutamate receptors, which are present not only in neurons but also in immunoreactive cells, neutrophils, erythrocytes, cardiomyocytes, and osteoblasts. Activation of these cells by HCY leads to increased cytoplasmic calcium ions, reactive oxygen species accumulation and MAP kinase activation. The overload of immunoreactive cells activates necrotic and apoptotic cell death^26^.

Inflammatory determinant clusters that are affected by high HCY levels include adhesion molecules, endothelial dysfunction, oxidative stress, leukocyte adhesion, and reduced NO bioavailability^27^. As a consequence, the vagina will becomes less immunological, making individuals who have high HCY more prone to infection.

Our research has some drawbacks. First, the cross-sectional design precluded us from establishing directionality or causality. The results may continue to be influenced by some additional unmeasured variables even after many adjustments. Second, although a sizable sample was used, only people who lived in the United States were included in the study. As a result, it must be taken into account when extrapolating to other populations. Therefore, to support our findings, high-quality multicenter controlled trials are needed.

## Conclusions

This study found a linear positive relationship between HCY and BV at a narrow 95% CI interval. This study provides some data support for clinical practice, and randomized controlled studies need to be used to provide more evidence support. Based on our results, we found that BV—positive individuals were more likely to have high BMI, RBC folate, ferritin and high HCY levels, Therefore, in clinical treatment, individuals with high BMI and those with HHCY need to pay great attention to whether they are at risk of developing BV and should be immediately examined and treated as soon as the risk appears. We were able to guide reminders to the clinical attention of the risk of crossover of the above two disorders in patients with BV and HHCY, respectively, when we obtained a positive relationship between BV and HCY. This is one of the clinical implications of our study.

### Supplementary Information

Supplementary Table S1.

Supplementary Table S2.

## Data Availability

Original data generated and analyzed during this study are included in this published article or in the data repositories listed in References. The dataset supporting the conclusions of this article is available in the NHANES repository, https://www.cdc.gov/nchs/nhanes/index.htm.
