# Supplementary material for: The association between homocysteine and bacterial vaginosis: results from NHANES 2001–2004
Source: Sci Rep. 2023 Dec 4;13:21388. doi: 10.1038/s41598-023-45494-5 (PMC10695932; doi:10.1038/s41598-023-45494-5)
Supplement: Supplementary file 1 — Supplementary Table S1. [file 41598_2023_45494_MOESM1_ESM.docx]

**Table S1 Standardized β of all covariates (predictors) in the fully adjusted model for prediction of Bacterial Vaginosis(BV)**

| Covariates | Standardized β | Unstandardized β (95%CI) | P | Collinearity statistics (VIF) |
| --- | --- | --- | --- | --- |
| Age | 0.0023 | 1.0023(0.9949,1.0098) | 0.5441 | 1.5 |
| Race |  |  |  | 1.1 |
| Mexican American(Reference) | - | - | - |  |
| Other Hispanic^#^ | 0.1908 | 1.2102(0.7871,1.8610) | 0.3847 |  |
| Non-Hispanic White^#^ | -0.7001 | 0.4965(0.4025,0.6126) | <0.0001 |  |
| Non-Hispanic Black^#^ | 0.5781 | 1.7827(1.4250,2.2303) | <0.0001 |  |
| Other Race - Including Multi-Racial^#^ | -0.1992 | 0.8194(0.5199,1.2916) | 0.3909 |  |
| Education level |  |  |  | 1.3 |
| <High school(Reference) | - | - | - |  |
| High school^##^ | -0.0486 | 0.9525(0.7635,1.1883) | 0.6664 |  |
| >High school^##^ | -0.3419 | 0.7104(0.5926,0.8516) | 0.0002 |  |
| BMI | 0.0348 | 1.0354(1.0237,1.0473) | <0.0001 | 1.1 |
| folate, rbc (ng/ml rbc) | -0.0033 | 0.9968(0.9959,0.9976) | <0.0001 | 1.2 |
| vitamin b12, serum (pg/ml) | 0.0001 | 1.0001(0.9999,1.0002) | 0.3170 | 1 |
| folate, serum (ng/ml) | -0.0474 | 0.9537(0.9395,0.9681) | <0.0001 | 1.1 |
| ferritin (ng/ml) | 0.0019 | 1.0019(1.0005,1.0033) | 0.0083 | 1.1 |
| segmented neutrophils percent (%) | -0.0151 | 0.9850(0.9769,0.9931) | 0.0003 | 2.6 |
| segmented neutrophils number | -0.0154 | 0.9847(0.9452,1.0259) | 0.4612 | 2.7 |

# Compared with Mexican American

## Compared with <High school

Note: β = beta (regression coefficients); BMI = Body Mass Index; CI = confidence intervals; VIFs = Variance Inflation Factors.
