# Supplementary material for: The association between homocysteine and bacterial vaginosis: results from NHANES 2001–2004
Source: Sci Rep. 2023 Dec 4;13:21388. doi: 10.1038/s41598-023-45494-5 (PMC10695932; doi:10.1038/s41598-023-45494-5)
Supplement: Supplementary file 2 — Supplementary Table S2. [file 41598_2023_45494_MOESM2_ESM.docx]

**Table S2 Analysis of the threshold effect of Homocysteine(HCY) on the prevalence of Bacterial Vaginosis(BV)**

|  | Crude Model | | Adjusted 1 | | Adjusted 2 | |
| --- | --- | --- | --- | --- | --- | --- |
| Homocysteine | OR for BV (95%CI) | P-value | OR for BV (95%CI) | P-value | OR for BV (95%CI) | P-value |
| Model Ⅰ |  |  |  |  |  |  |
| A straight line effect | 1.10 (1.06, 1.14) | <0.0001 | 1.11 (1.06, 1.15) | <0.0001 | 1.1 (1.0, 1.1) | <0.001 |
| Model Ⅱ |  |  |  |  |  |  |
| Inflection point(K) | 8.28 | | 5.8 | | 8.2 |  |
| <K-segment effect 1 | 1.07 (1.01, 1.14) | 0.0231 | 1.17 (1.02, 1.34) | 0.0215 | 1.0 (1.0, 1.1) | 0.356 |
| >K-segment effect 2 | 1.13 (1.05, 1.22) | 0.0017 | 1.09 (1.04, 1.15) | 0.0006 | 1.1 (1.0, 1.2) | 0.005 |
| Log Likelihood Ratio Tests | 0.371 | | 0.386 | | 0.196 | |

Non-adjusted model adjust for: None
Adjust 1 for: Age, Educational level, Race, BMI

Adjust 2 for: Adjust 1 + rbc folate, serum folate
